# Janus-structured Reg/PVA/PAN@TiO_2_ nanofiber dressing containing RegIIIγ recombinant antimicrobial peptides for promoting wound healing

**DOI:** 10.1039/d5ra05169j

**Published:** 2025-11-17

**Authors:** Xuewei Fu, Jianrong Chen, Xuewen Jian, Junkai Wang, Minjian Liao, Pin Xiong, Xiaochun Wu, Yuqiao Liu, Xianming Dong, Wuyi Zhou, Hui Zhao

**Affiliations:** a Key Laboratory for Biobased Materials and Energy of Ministry of Education, Research Center of Biomass 3D Printing Materials, School of Materials and Energy, South China Agricultural University Guangzhou 510642 P. R. China dongxming@263.net zhouwuyi@scau.edu.cn totom2008@scau.edu.cn; b Guangdong Lingnan Health Ecology Technology Group Co., Ltd Jiangmen 529100 P. R. China 75843056@qq.com; c School of Veterinary Medicine, South China Agricultural University Guangzhou 510642 P. R. China

## Abstract

In clinical practice, the development of advanced wound dressings capable of simultaneously preventing infection and promoting healing remains a critical challenge. To address this, we engineered a Janus-structured nanofiber dressing RegIIIγ/polyvinyl alcohol/polyacrylonitrile@titanium dioxide (Reg/PVA/PAN@TiO_2_) *via* electrospinning, incorporating the recombinant antimicrobial peptide RegIIIγ—obtained through genetic recombination—which exhibits dual functionality by preventing infection and accelerating wound healing. The bilayer architecture consists of a hydrophilic polyvinyl alcohol layer (Reg/PVA) for sustained release of RegIIIγ to enhance therapeutic utilization, and a hydrophobic polyacrylonitrile/TiO_2_ (PAN@TiO_2_) outer layer for barrier protection. The morphology, chemical structure, and thermal stability of the dressing were characterized, and the antibacterial activity, cell compatibility, and wound healing efficacy were evaluated through *in vitro* cell experiments and *in vivo* animal models, elucidating the wound healing mechanism of Reg/PVA/PAN@TiO_2_. The results demonstrated that the Reg/PVA/PAN@TiO_2_ nanofiber dressing exhibited excellent antibacterial properties (33.6 mm), robust mechanical performance (2.5 MPa) and biocompatibility with L929 fibroblasts (>80%). The dressing also significantly accelerated the wound healing process of full-thickness skin defects, reduced inflammatory responses, and promoted collagen synthesis and tissue regeneration. The healing speed and efficacy were significantly superior to those of the RegIIIγ drug alone. Therefore, the Reg/PVA/PAN@TiO_2_ dressing, with its unique Janus design, potent antibacterial activity and superior pro-healing properties, has significant potential for clinical application in the management of infected wounds. It also offers a novel strategy to overcome the drawbacks of antimicrobial peptides, such as rapid degradation, short half-life and limited clinical efficacy.

## Abstract 2

In clinical practice, the development of advanced wound dressings capable of simultaneously preventing infection and promoting healing remains a critical challenge.

## Graphical abstract

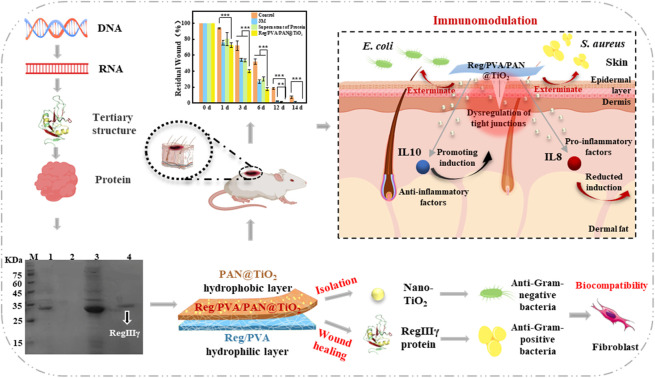

## 1. Introduction

The key to wound care lies in the prevention and control of bacterial infections. Silver-containing wound dressings are widely used in clinical settings, with a market share of 70%, however the potential risks associated with heavy metals remain a focal point of concern.^1,2^ Long-term use of antibiotics in wound dressings can lead to the emergence of resistant bacterial strains or cause allergic reactions. In contrast, antimicrobial peptides (AMPs), which are natural host defense factors found in plants, animals and microorganisms, offer several advantages.^3,4^ They not only possess low molecular weight, specific mechanisms of action and broad-spectrum antimicrobial activity but also promote cell proliferation and growth, facilitate wound healing, regulate hormones, and play significant roles in immune modulation.^5–8^

RegIIIγ, derived from the mouse intestine, belongs to the C-type lectin family of regenerating islet-derived proteins.^9–13^ Studies have shown that it has broad-spectrum resistance, low resistance development, regulation of host inflammatory responses, biofilm combatting capabilities, and rapid action.^14,15^ Additionally, the degradation products of peptide drugs are natural amino acids, posing low metabolic risks and being safer for the human body. The antimicrobial mechanism of RegIIIγ primarily involves the specific recognition of peptidoglycan in the cell walls of Gram-positive bacteria,^16–18^ leading to the destruction of bacterial cell walls, killing Gram-positive bacteria, and forming a sterile isolation zone between exotoxins and the epithelium. This makes it difficult for bacteria to develop resistance due to the specific antimicrobial action of AMPs.^19,20^ However, due to the small molecular weight, easy degradation, and short half-life of AMPs, their clinical application remains challenging. Enhancing the biostability and utilization of AMPs is the main issue our study aims to address.

Normal skin exhibits a three-dimensional layered structure from outer to inner: epidermis – dermis – subcutaneous tissue. Together, these layers maintain the skin's barrier protection, material exchange, and wound repair capabilities, providing the core basis for biomimetic design in wound dressings.^21^ Janus-structured materials, due to their unique asymmetric hydrophilic and hydrophobic properties, show great potential in the fields of energy, biomedicine and smart textiles, and represent a key material tool for the interdisciplinary intersection of chemistry, physics, materials and life.^22–28^ Janus-structured materials employ a “functional zone design” to specifically address the challenges of full-thickness wound repair. They mimic the epidermal layer's function to provide “passive protection + active barrier” at the surface, while emulating the dermal layer's function to deliver “ECM biomimicry + active repair” at the deeper layers. This resolves the core contradiction that “a single dressing cannot meet multiple needs simultaneously.” Electrostatic spinning technology, as the core method for producing Janus nanofibers, has made significant progress in recent years in terms of synthesis strategy, functionalization design and application expansion.^29–36^ However, it still faces many challenges, such as the low mechanical strength of biomedical Janus hydrogels (*e.g.* tensile strength <1 MPa) and the susceptibility of long-term antimicrobial activity to decline due to non-uniform drug retardation.

In this study, a Janus-structured nanofiber membrane Reg/PVA/PAN@TiO_2_ carrier with both hydrophilic and hydrophobic properties was developed. RegIIIγ is incorporated into the network structure of the PVA polymer to form a hydrophilic layer (Reg/PVA) capable of slowing down the release and half-life of the RegIIIγ protein drug, which plays an important role in wound healing. The combination of PAN and nano-TiO_2_ creates an external hydrophobic matrix while maintaining good water vapor transmission rate (WVTR).^37–42^ This not only effectively prevents external infections but also creates a comfortable and favorable microenvironment for the regeneration of skin tissue. Additionally, it fills the hydrophilic framework of Reg/PVA, facilitating the proliferation and aggregation of fibroblasts on the surface of the Reg/PVA/PAN@TiO_2_ nanofiber dressing, thereby accelerating wound healing. These finding establish a new paradigm for developing AMP-based dressing with potential for clinical application.

## 2. Materials and methods

### 2.1 Materials and reagents

Polyvinyl alcohol (PVA) (*M*_w_ 130 000 Da), polyacrylonitrile (PAN, *M*_w_ 50 000 Da), nano-TiO_2_ and *N*,*N*-dimethylformamide (DMF, 99%) were supplied by Sigma-Aldrich (Shanghai, China); Tris–HCl (10 mM, pH = 8.5), NaCl, Triton X, EDTA, imidazole and guanidine hydrochloride were purchased from Genview (Fuzhou, China); Dulbecco's modified Eagle's medium (DMEM), fetal bovine serum (FBS), trypsin, penicillin–streptomycin (P/S) and phosphate-buffered saline (PBS, 10×, pH = 7.4) were obtained from Servicebio (WuHan China). MaximaTMSYBRGreen/ROXqPCRMasterMix (2×) (Cat. no. #A25742), reverse transcription kit (K1622) were supplied by Thermo Fisher Scientific; total RNA extraction kit was purchased from Promega (Shanghai, China); fifty healthy 6–8 weeks old SPF male KM mice (weight 25–35 g).

### 2.2 Preparation and characterization of mouse-derived antimicrobial peptide RegIIIγ

#### 2.2.1 PCR amplification and gel electrophoresis of DNA

The recombinant plasmid pET3a-*RegIIIγ* was used as a template, with the specific primers 5′-GGAATTCATGAGCAGCTGCCCCAA-3′ (forward primer) and 5′-CCGCTCGAGCTAGGCCTTGAATTT-3′ (reverse primer). The forward primer included an *EcoR*I restriction site for cloning into pET32a, while the reverse primer incorporated the native stop codon followed by an *Xho*I site. PCR products were amplified using a Bio-Rad T100 thermal cycler (Thermo Fisher, China), identified and subsequently purified and recovered from agarose gels using the SteadyPure Agarose Gel DNA/PCR product small volume recovery Kit (Accurate biology, ChangSha, China). Both PCR products and the vector were digested with *EcoR*I and *Xho*I, gel-purified, and ligated. The recombinant plasmid pET32a-*RegIIIγ* was then transformed into *E. coli* BL21-(DE3) for protein expression.

#### 2.2.2 The expression of RegIIIγ peptide

Activated *E. coli* BL21-(DE3) harboring pET32a-*RegIIIγ* vector were cultured at a ratio of 1/50 in 500 mL of LB medium supplemented with 0.1 mg mL^−1^ ampicillin for 3 hours at 37 °C. Protein expression was induced by the addition of 0.4 mM IPTG (Genview, Fuzhou, China), followed by an additional 3 hours incubation at 37 °C with good aeration. *RegIIIγ* bacterial liquid was incubated for another 3 h at 37 °C with good aeration. The Cells were harvested by centrifugation at 10 000*g* for 10 min at 4 °C, and the pellet was resuspended in 1/10th of the culture volume (50 mL) of Buffer I (50 mM Tris, 0.15 M NaCl, 1% Triton X-100, pH 8.0). Cells were ruptured by sonication for 20 min, with 3 seconds pulses at 8 seconds intervals. The lysate was then centrifuged at 10 000*g* for 20 min, and the insoluble fraction was resuspended in 50 mL of Buffer II (50 mM Tris, 150 mM NaCl, 2 mM EDTA, 20 mM imidazole, 1% Triton X-100, pH = 8.0). This centrifugation and resuspension process was repeated, add 1 mL of Buffer A (1 M Tris, 1 M imidazole, pH 8.0) was added to the insoluble inclusion body to ensure its dissolution. The inclusion body was resuspended in 20 mL of Buffer III (50 mM Tris, 0.15 M NaCl, 8 M guanidine hydrochloride, pH = 8.0). Following another round of centrifugation and resuspension, the final supernatant obtained was then repeated by centrifugation, the purified protein was subsequently identified by SDS-PAGE.

#### 2.2.3 BCA assay

The protein concentration of the supernatant and sediment were determined using bicinchoninic acid assay (BCA, DINGGUO Biotechnology, Beijing, China). A standard curve was plotted to determine the sample concentrations.

#### 2.2.4 Antibacterial activity *in vitro*

The Gram-positive bacteria *Staphylococcus aureus* ATCC 12600 (*S. aureus*) was cultured in LB liquid medium. The antibacterial ability of these samples was evaluated using the bacterial inhibition zone assay. LB liquid medium containing *S. aureus* (10^6^ CFU per mL; harvested at the log growth phase) was evenly spread on agar plates. Subsequently, 100 µL of the sample, 10 µL of ampicillin (100 mg mL^−1^) or a 10 mm nanofiber film was positioned on the agar plates and incubated at 37 °C overnight. The average diameter of the inhibition zones was measured to assess the antibacterial activity.

### 2.3 Preparation and characterization of Reg/PVA/PAN@TiO_2_ wound dressings

#### 2.3.1 Preparation and characterization of the Reg/PVA hydrophilic layer

To prepare a 12% (w/v) polyvinyl alcohol (PVA) spinning solution, meanwhile, the supernatant of the purified recombinant protein RegIIIγ is lyophilized and then accurately incorporated into 20 mL of electrospinning solution at proportions of 1%, 3%, 6%, 8%, and 10% (wt%), respectively, resulting in five different concentrations of Reg/PVA mixtures. After ultrasonically crushing the mixtures until completely dissolved, electrospun nanofibers are prepared using an electrospinning device (Yongkang Leyet Technology Development Co., Ltd SS-3556H, Beijing, China). The specific spinning parameters are as follows: needle size, 23 G; flow rate, 0.1 mL h^−1^; voltage, 23.0 kV; distance between the tip of the spinneret and the drum collector, 5 cm; collector speed, 80% of the rolling speed. Electrospinning continues for up to 18 hours at an ambient temperature of approximately 28 °C and a relative humidity range of 45–50%. The morphology characteristics (SEM, JSM-7001F, JEOL, Japan) of the Reg/PVA hydrophilic layers with different proportions are examined, along with their tensile properties (Tensile tester, 5542A, Instron, USA) and antibacterial capabilities, to determine the optimal incorporation ratio of the RegIIIγ protein is 6%.

#### 2.3.2 Preparation of Reg/PVA/PAN@TiO_2_ nanofiber dressing

**Fig. 1:**
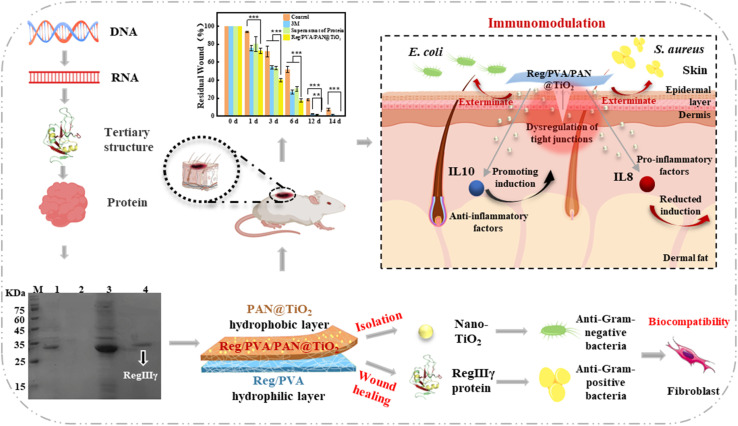
Schematic of the Reg/PVA/PAN@TiO_2_ nanofiber dressing production, composition and function.

Polyacrylonitrile (PAN) was dissolved in dimethylformamide (DMF) to produce a 10% (w/v) PAN solution. Then nano-TiO_2_ (0.2 wt%) was added to the PAN spinning solution, and stirred with a magnetic stirrer at 45 °C for 30 min, followed by 20 min of ultrasonication to form a homogenous spinning solution. Subsequently, electrospinning the PAN spinning solution containing nano-TiO_2_ onto the 6% Reg/PVA nanofiber membrane to obtain the recombinant murine-derived antimicrobial peptide RegIIIγ composite nano-biological antimicrobial dressing, referred to as Reg/PVA/PAN@TiO_2_, as shown in Fig. 1. The electrospinning parameters were as follows: needle size, 22 G, flow-rate 0.3 mL h^−1^; voltage, 17 kV; distance between the tip of the spinneret and the drum collector, 8 cm; and collector speed, with the drum rolling at 80%. Electrospinning was continued for up to 2 h at ambient temperature (∼30 °C) and relative humidity ranging from 45% to 50%. The tensile strength of the dressing was measured by Universal Testing Machine (INSTRON 5565, USA), and the morphology was assessed by field emission scanning electron microscopy (SEM, JSM-7001F, JEOL, Japan).

#### 2.3.3 Water contact angle

The water contact angle (WAC) of the scaffolds was measured using the contact angle instrument (FSF003, AFES, China) with a 3 µL liquid droplet.

#### 2.3.4 Infrared spectroscopy

A Bruker Tensor Fourier Transform Infrared (FTIR) spectrometer (Bruker, Germany) was used to obtain the infrared absorption spectra of the samples. The spectra were recorded at a resolution of 2 cm^−1^ in the range of 4000–600 cm^−1^, with a total of 32 scans.

#### 2.3.5 Thermogravimetric analysis

The thermogravimetric analysis (TGA) of the wound dressings was carried out by using a Thermogravimetric Analyzer (PerkinElmer Enterprise Management (Shanghai) Co., Ltd, TGA8000, Shanghai, China).

#### 2.3.6 Water vapor transmission rate (WVTR)

To determine the moisture permeability of the film, the amount of moisture that penetrated an area of 33.18 cm^2^ was measured under the conditions of 90% RH, 38 °C temperature, and 60 min test interval using a W3/031 (Labthink, Medford, MA, USA), in line with the ASTM E96 standards.

#### 2.3.7 Biocompatibility of wound dressings *in vitro*

Adult fibroblasts (L929) were cultured with medium (DMEM), and inoculated in 96-well cell plates. The plates were incubated at 37 °C with 5% CO_2_ until the cells were confluent. The test material was added and cultured in the incubator for 24, 48 and 72 h. At each time point, cell proliferation was assessed using the cell counting kit-8 (CCK-8, C0038, Beyotime, China). The cells were incubated for 2 h at 37 °C and the absorbance at 450 nm was measured. Cell viability and death were assessed using AM/PI kit (Ding Guo, Bei Jing, China) after incubation at 37 °C for 30 min, and the staining effect was observed under a fluorescence microscope and photographed.

### 2.4 Analysis of wound healing

#### 2.4.1 Wound healing model

The Reg/PVA/PAN@TiO_2_ nanofiber dressing was evaluated in a mouse dermal wound model. Fifty KM mice (female or male, 6–8 weeks, weight 25–35 g) were housed in a facility with controlled humidity (40–70%) and temperature (23 ± 2 °C) under specific pathogen-free conditions. The animal procedures were approved by the Ethical Committee on Animal Care and Use of South China Agricultural University, China (ethics clearance: 2023f222). All animal experiments were performed in accordance with China's guidelines and laws for animal protection.

Before the wound healing operation, the mice were anesthetized with injection the hair on their backs was shaved to expose the skin. After sterilization, one full-thickness circular skin wounds (1 cm in diameter) were created using a surgical scalpel and randomly divided into four categories, with 12 (male mice) in each group: Reg/PVA/PAN@TiO_2_ nanofiber dressing, supernatant of purified protein RegIIIγ, 3 M wound dressings (3 M™, Shang Hai, China), and untreated control groups (wounds only). Only one change of medication was performed on the second day, with no further changes thereafter. Following surgery, the mice were caged and monitored daily. Digital photographs were used to monitor the wound closure over time and were taken on different days after wounding. A ruler was placed adjacent to the wound to provide a reference for measuring the area of the wound. Photographs were analyzed and the wound area was determined.

#### 2.4.2 Histopathological analysis

Animals were euthanized at different time points and the wound and organ tissues were harvested. Specimens were fixed in 4% paraformaldehyde for 24 h followed by PBS washing and incubation in 70% (v/v) ethanol until further analysis. The specimens were dehydrated with a graded ethanol series and embedded in paraffin. Then, 8 µm thick sections were obtained and analyzed by using hematoxylin and eosin (H&E) and Masson's trichrome staining.

#### 2.4.3 Serum biochemical analysis

At the end of this experiment, blood samples from the mice were collected *via* ocular blood collection prior to euthanasia. Serum was immediately separated by centrifugation at 3000 rpm for minutes at 4 °C. The obtained serum samples were stored at −80 °C for blood biochemical analysis. Serum biochemical indexes were measured using an animal biochemical analyzer (SMT-120VP, Seamaty, CHINA) and an animal biochemical reagent plate (Seamaty, CHINA). The concentrations of various biochemical markers were determined, including liver function markers: alanine transaminase (ALT), alkaline phosphatase (ALP), aspartate aminotransferase (AST), total protein (TP), albumin (ALB), total bilirubin (TBIL), renal function: creatinine (CREA), uric acid (UA); and cardiac enzymes: lactate dehydrogenase (LDH), creatine kinas (CK).

#### 2.4.4 Quantification of inflammatory factor expression by qPCR

Total RNA was isolated from wound skin tissue using a total RNA extraction kit (Promega, Shanghai, China). Reverse transcription to obtain complementary DNA (cDNA) was performed from total RNA with oligo (dT) primer and a reverse transcription kit (Thermo, Shanghai, China); cDNA samples were stored at −20 °C until use. Quantification of gene expression of inflammatory factors IL8 and IL10 was performed by the real-time quantitative PCR (qPCR) using the Step One QPCR instrument (Applied Biosystems, Shanghai, China). Primers were obtained from National Center for Biotechnology Information platform, IL8: NM_011339.2, IL10: NM_010548.2 and β-Actin (NM_007393.5) as a reference gene. The results obtained were analyzed by the 2^−Δ*C*_q_^ comparison method.

### 2.5 Statistical analysis

The statistical analysis was conducted using analysis of variance (ANOVA) with SPSS software. A *p*-value below 0.05 was considered statistically significant.

## 3. Results and discussions

### 3.1 Preparation and characterization of RegIIIγ protein

**Fig. 2:**
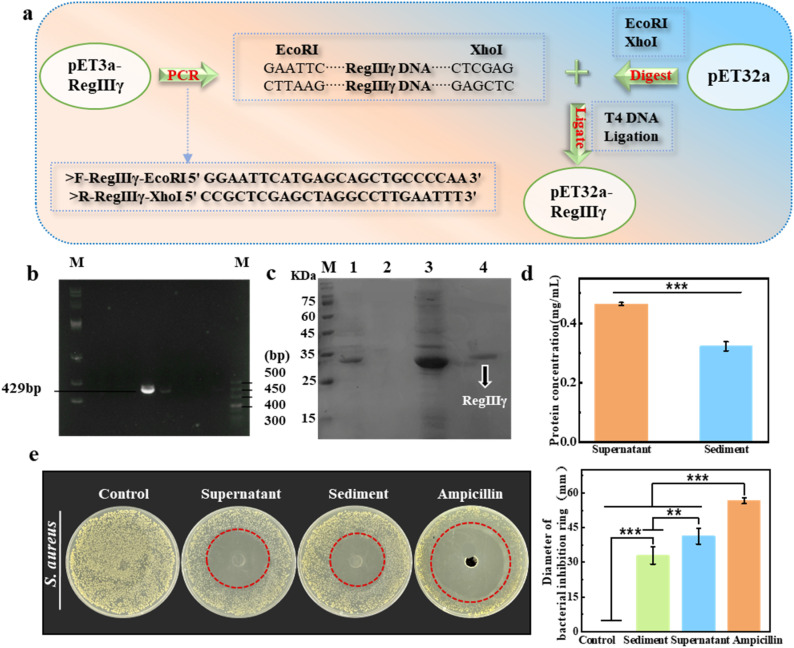
(a) Schematic diagram of the construction of recombinant plasmid pET32a-*RegIIIγ*; (b) PCR product agarose gel electrophoresis; (c) M: protein marker; lane 1: supernatant obtained after centrifugation following Buffer I treatment; lane 2: supernatant obtained after centrifugation following Buffer II treatment; lane 3: sediment obtained after centrifugation following Buffer II treatment; lane 4: final supernatant obtained after Buffer III treatment. (d) Concentration of supernatant and sediment in the purified protein; (e) antibacterial activity and diameter of the bacterial inhibition zone of the supernatant and sediment of purified protein against *S. aureus*.

According to the construction process of the pET32a-*RegIIIγ* recombinant plasmid vector (Fig. 2a and S1) the target gene *RegIIIγ* (429bp) was first obtained through PCR and then ligated with the pET32a vector to construct the recombinant plasmid pET32a-*RegIIIγ*. After induction, the recombinant protein RegIIIγ (15.6 kDa) was expressed as a fusion protein with the TrxA tag (11.9 kDa) on the pET32a vector to form a recombinant protein (27.5 kDa) (Fig. 2b, c and S2). The TrxA tag is a thioredoxin that facilitates the formation of disulfide bonds in the exogenous gene, thereby enhancing the solubility, stability, and simplifying the extraction and purification of the fusion protein.

The concentration of RegIIIγ in the cell lysate supernatant and precipitate was measured using a BCA protein assay (Fig. S3). The results showed that the concentration of the protein in the supernatant was slightly higher than that in the sediment (Fig. 2d). Further studies on the antibacterial effects of the supernatant and sediment were conducted *via* a spreading plate method, with taking Gram-positive *S. aureus* as the test bacteria. The related results are exhibited in Fig. 2e. In comparison, the inhibitory effect on bacterial colonies by the supernatant and sediment was significantly different from the control group, indicating that the obtained recombinant protein RegIIIγ has certain bactericidal effects. Moreover, the diameter of the inhibition zone of the supernatant against *S. aureus* was almost comparable to that of ampicillin, demonstrating the excellent antimicrobial capability of the recombinant RegIIIγ against *S. aureus*.

### 3.2 Characterization of Reg/PVA/PAN@TiO_2_ nanofiber dressing

#### 3.2.1 The structure and interfacial characteristics of Reg/PVA/PAN@TiO_2_ nanofiber dressing

**Fig. 3:**
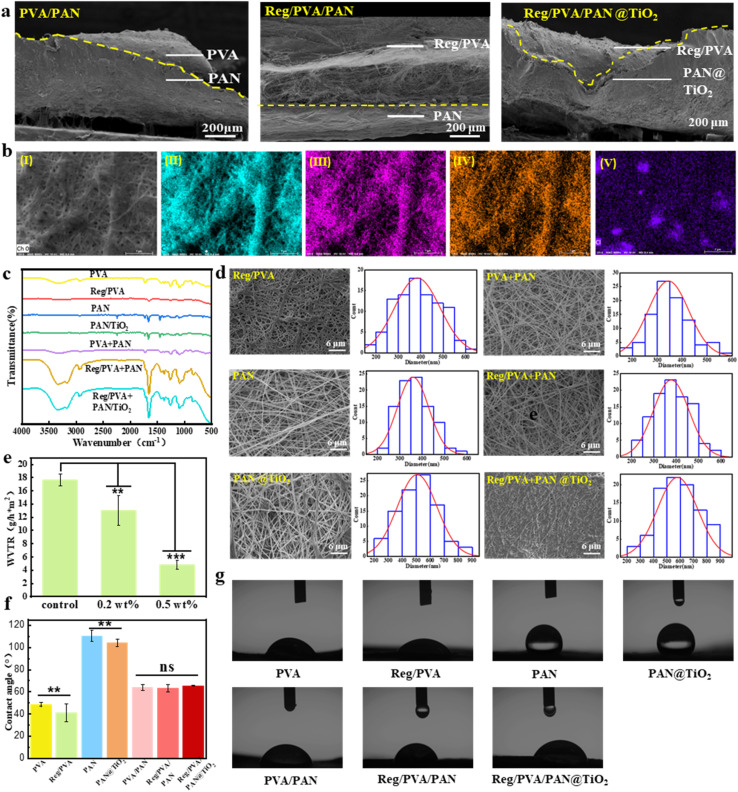
(a) Cross-sectional structures of nanofiber dressing; (b) SEM-mapping. (I) SEM images of Reg/PVA, (II) C, (III) O, (IV) N, (V) Cl; (c) FTIR spectra for nanofiber dressing; (d) SEM images of the wound dressings. (e) Comparison of water vapor transmission rate (WVTR). (f) Water contact angle measurements. (g) Water contact angle measurement images.

The hierarchical architecture of Reg/PVA/PAN@TiO_2_ is illustrated in Fig. 1 and 3a. SEM elemental mapping (Fig. 3b) reveals uniform distributions of elements C, N, O, and Cl across the membrane, confirming the successful integration of RegIIIγ with PVA. Localized Cl-rich clusters may originate from residual buffer salts. FTIR analysis (Fig. 3c) further verifies the effective loading of RegIIIγ protein: distinct peaks at about 1600 cm^−1^ (amide I, C=O stretching vibration) and about 1200 cm^−1^ (amide III, N–H bending vibration) in the Reg/PVA layer indicate preserved α-helix and β-sheet conformations of the protein. Notably, no characteristic peaks corresponding to Ti–O–C bonds were observed in the PAN@TiO_2_ spectrum. These results suggest that TiO_2_ nanoparticles are physically adsorbed onto the PAN fiber surface without chemical bonding.

As shown in Fig. S4, increasing the RegIIIγ concentration leads to enlarged fiber diameters and observable protein aggregation on the nanofibrous wound dressings, suggesting that drug-loading capacity can be modulated by optimizing electrospinning parameters. Based on comprehensive evaluations of drug-loading capacity, mechanical properties (Fig. S5), and antibacterial performance (Fig. S6), the optimal RegIIIγ concentration in the Reg/PVA layer was determined to be 6%.

Contact angle measurements (48°, Fig. 3f and g) demonstrated substantial hydrophilicity for the 6% Reg/PVA surface. Such hydrophilicity enables immediate dissolution upon hydration for controlled protein release, while the microporous structure (Fig. 3d) facilitates exudate absorption and maintenance of a moist microenvironment, which is critical for cell proliferation and granulation tissue formation. In contrast, the PAN@TiO_2_ outer layer exhibits significant hydrophobicity (contact angle: 112°, Fig. 3g and f) and large pore size and porosity (Fig. 3d), which supports efficient water vapor transmission (WVTR, Fig. 3e). This design prevents exudate accumulation while maintaining aerobic conditions for tissue repair. The incorporation of TiO_2_ nanoparticles enhances the physical barrier against external contaminants. Optimizing the TiO_2_ content to 0.2 wt% (WVTR: 12.99 g m^−2^/24 h, Fig. 3e) achieves a balance between antimicrobial efficacy and breathability, superior to conventional hydrophobic dressings prone to hypoxia-induced delayed healing.

Structurally, the Janus-like configuration of Reg/PVA/PAN@TiO_2_ (Fig. 3a) utilizes interfacial interactions between dissimilar polymers to establish gradient porosity: the dense Reg/PVA sublayer restricts fibroblast overgrowth, whereas the macroporous PAN@TiO_2_ overlayer promotes gas exchange.^43,44^ This pore architecture addresses the conflicting demands in chronic wound management—blocking microbial invasion while enabling metabolic waste clearance.

Functionally, the Reg/PVA/PAN@TiO_2_ bilayer design overcomes the limitations of single-layer nanofibrous systems by decoupling drug delivery and barrier functions. The hydrophilic layer ensures rapid therapeutic action *via* immediate drug release, while the hydrophobic layer provides sustained protection—a temporal synergy critical for managing infected wounds. This dual-functionality highlights the potential of Janus-structured dressings in advanced wound care applications.

#### 3.2.2 The *v* and thermal stability of the Reg/PVA/PAN@TiO_2_ nanofiber dressing

**Fig. 4:**
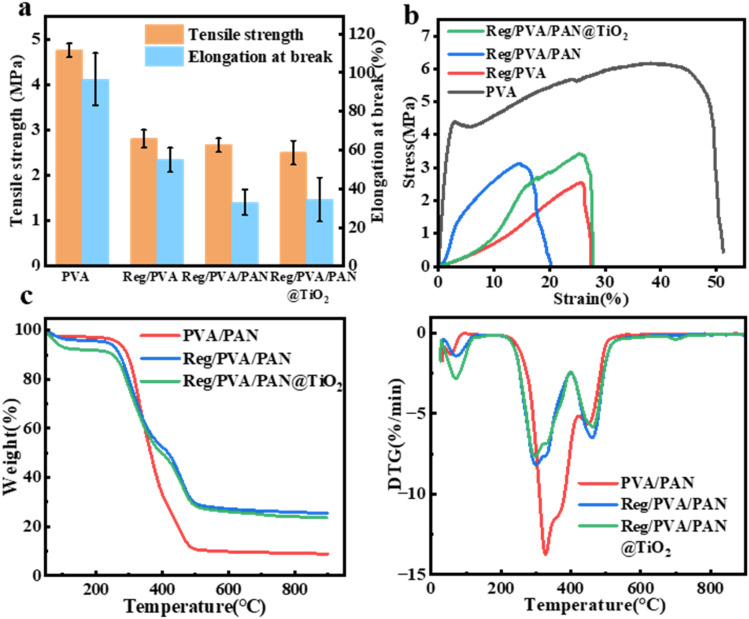
(a) Tensile properties of Reg/PVA/PAN@TiO_2_; (b) tensile stress–strain curves of Reg/PVA/PAN@TiO_2_; (c) TG curves of nanofiber wound dressings.

The mechanical performance of nanofibrous membranes is a critical determinant of their practicality as wound dressings. As illustrated in Fig. 4a, the mechanical properties of Reg/PVA membranes gradually declined with increasing RegIIIγ protein concentration compared to pure PVA nanofibers (Fig. S4 and 4a). This phenomenon can be attributed to the introduction of RegIIIγ, a small-molecule antimicrobial peptide, which disrupts hydrogen bonding interactions between PVA molecular chains, resulting in structural loosening within the membrane. Notably, although the tensile strength of 6% Reg/PVA decreased by 35% relative to pure PVA, it remained significantly higher than the minimum clinical requirement for wound dressings. Upon further incorporation of the PAN@TiO_2_ layer, the mechanical performance of the Reg/PVA/PAN@TiO_2_ composite membrane showed no significant deterioration compared to Reg/PVA. Intriguingly, the stress–strain curve revealed an enhanced elongation at break for Reg/PVA/PAN@TiO_2_, matching the level of Reg/PVA (Fig. 4b). This improvement suggests that the physical doping of TiO_2_ nanoparticles compensates for the reduced ductility induced by PAN through interface enhancement effects. Collectively, the tensile strength of Reg/PVA/PAN@TiO_2_ (2.5 MPa) was mechanically comparable to previously reported materials such as ε-PL/PCL/Gel (2.5 MPa) and PCL/PEG/*H. perforatum* oil (2.5 MPa) (Table S1), validating its mechanical feasibility as a functional dressing. The tensile strength of Reg/PVA/PAN@TiO_2_ falls significantly within the average range of natural skin (2.5 MPa *vs.* 2–8 MPa), meeting the mechanical support requirements for minor wounds without tearing during use.^45^ The TGA curves (Fig. 4c and S7) further elucidate the thermal degradation behavior of the materials. Pure PVA exhibited rapid weight loss (80%) in the 250–300 °C range due to backbone scission, while Reg/PVA/PAN@TiO_2_ showed a significantly lower weight loss rate (60%) in the same temperature interval. Comparative analysis of DTG profiles for PVA/PAN, Reg/PVA/PAN and Reg/PVA/PAN@TiO_2_ revealed slower decomposition rates for the latter two systems. This enhanced thermal stability may arise from increased intermolecular cross-linking density induced by RegIIIγ protein incorporation, which delays degradation kinetics. These results confirm the superior thermal stability of Reg/PVA/PAN@TiO_2_, aligning with the barrier effect theory of nanocomposites.

### 3.3 The biological functions of Reg/PVA/PAN@TiO_2_ nanofiber dressing

#### 3.3.1 *In vitro* antibacterial properties

**Fig. 5:**
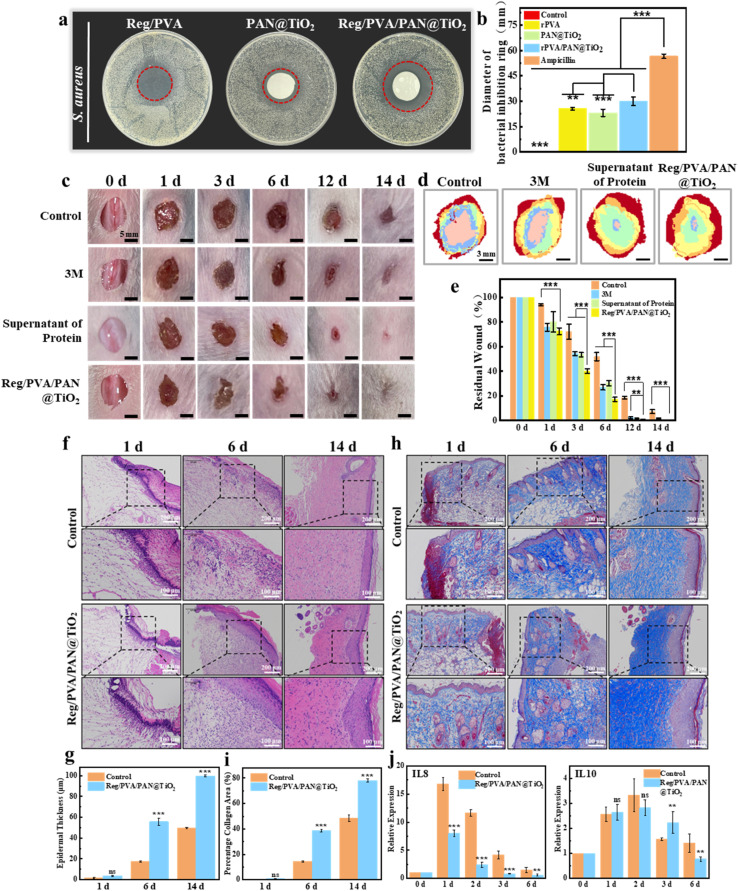
(a) Diameter of bacterial inhibition ring of the monolayer and nanofiber dressing; (b) antibacterial activity of the monolayer and nanofiber dressing; (c) view of wound healing at different time points. Scale bar: 2 mm; (d) wound healing boundaries at different time points in the various groups. The red area represents the initial wound area; while, the orange, yellow, green, blue and pink areas represent the wound area on days 1, 3, 6, 12 and 14, respectively. (e) Rate of residual wound healing. (f) HE staining images of skin at different time points during wound healing. (g) Wound healing epidermal thickness at different time points in the various groups. (h) Masson's staining images of skin at different time points during wound healing. (i) Wound healing quantitative analysis of collagen area at different time points in the various groups. (j) Expression levels of IL8 and IL10 during wound healing.

The antimicrobial efficacy of nanofibrous dressings serves as a critical determinant for their clinical applicability. Agar diffusion assays (Fig. S6) revealed that the incorporation of RegIIIγ protein significantly enhanced the antibacterial activity of Reg/PVA, demonstrating a dose-dependent bactericidal effect. Notably, the composite membrane Reg/PVA/PAN@TiO_2_ produced significantly larger inhibition zones than either the single-layer Reg/PVA or PAN@TiO_2_, indicating synergistic antibacterial action between RegIIIγ and TiO_2_ nanoparticles (Fig. 5a and b).

#### 3.3.2 Wound healing promotion and healing mechanism

To validate the practical wound-healing efficacy, a murine full-thickness skin defect model (*n* = 15) with a diameter of 10 mm wound on its back was established (Fig. 1). Quantitative analysis demonstrated that the Reg/PVA/PAN@TiO_2_ group exhibited significantly higher wound closure rates at all time points (0, 1, 3, 6, 12, and 14 days) compared to controls including 3 M positive control group and protein supernatant group (Fig. 5d and e), achieving complete wound closure with hair regeneration within 14 days (Fig. 5c). In contrast, the RegIIIγ protein supernatant group required repeated dosing and showed delayed healing kinetics, highlighting the superiority of sustained protein release from the nanofibrous matrix in accelerating tissue repair and enhancing clinical outcomes.

Histological analysis elucidated the underlying healing mechanisms. H&E staining of regenerated wound tissue (Fig. 5f) demonstrated that the Reg/PVA/PAN@TiO_2_ group developed uniform and dense epidermis and granulation tissue as early as day 6, whereas controls exhibited minimal regeneration. By day 14, although both groups achieved complete epithelialization, the Reg/PVA/PAN@TiO_2_ group displayed a more structured dermal layer with significantly improved epidermal thickness and complexity (Fig. 5g). Masson's trichrome staining further delineated collagen deposition dynamics (Fig. 5h and i): dense and well-organized blue-stained collagen fibers were observed in the Reg/PVA/PAN@TiO_2_ group by day 6, with collagen density and alignment regularity surpassing controls by day 14. These findings collectively confirm the material's ability to optimize extracellular matrix remodeling.

#### 3.3.3 Inflammatory cytokine profiling further elucidated the pro-healing mechanisms

IL-8, a potent neutrophil chemoattractant, rapidly accumulates during the initial inflammatory phase to recruit neutrophils for pathogen clearance, but excessive expression of pro-inflammatory chemokines such as IL-8 can impede healing progression. As healing advances, anti-inflammatory IL-10 levels gradually rise to suppress inflammation and promote tissue remodeling. Quantitative PCR (qPCR) analysis (Fig. 5j) revealed that the Reg/PVA/PAN@TiO_2_ group maintained lower IL-8 expression than controls throughout the healing process, while IL-10 concentrations were significantly elevated by day 3, suggesting that the dressing modulates inflammatory responses by attenuating hyperinflammation and promoting anti-inflammatory/repair balance. Thus, the synergistic combination of histological evidence (accelerated epithelialization) and molecular profiling (IL-10 upregulation) conclusively demonstrated the Reg/PVA/PAN@TiO_2_ nanofiber dressing's ability to reprogram the wound microenvironment.

Based on the above studies, we investigated the wound healing effects of Reg/PVA/PAN@TiO_2_ nanofiber dressing *in vivo* and *in vitro*, at the molecular, cellular, and tissue levels, elucidating the mechanisms by which they promote wound healing. A present a comprehensive evaluation of the Reg/PVA/PAN@TiO_2_ nanofiber dressing in terms of hydrophilicity or hydrophobicity, antimicrobial activity, cell viability, wound healing rate and tensile properties, compared with previously reported wound dressing (Table S1).^39,46–57^ It can be seen that Reg/PVA/PAN@TiO_2_ nanofiber dressing can reach almost 100% in antimicrobial activity, cell viability and wound healing rate. The Reg/PVA/PAN@TiO_2_ is a bilayer composite membrane with a hydrophilic PVA layer and a hydrophobic PAN layer, whereas most of the wound dressings in those references are only hydrophilic. Its tensile strength is similar to other wound dressings, approximately 2.5 MPa. In summary, these results confirmed that the Reg/PVA/PAN@TiO_2_ nanofiber dressing can effectively promote the healing of full-thickness skin defect wounds.

### 3.4 Biocompatibility and biosafety evaluation of Reg/PVA/PAN@TiO_2_ nanofiber dressing

**Fig. 6:**
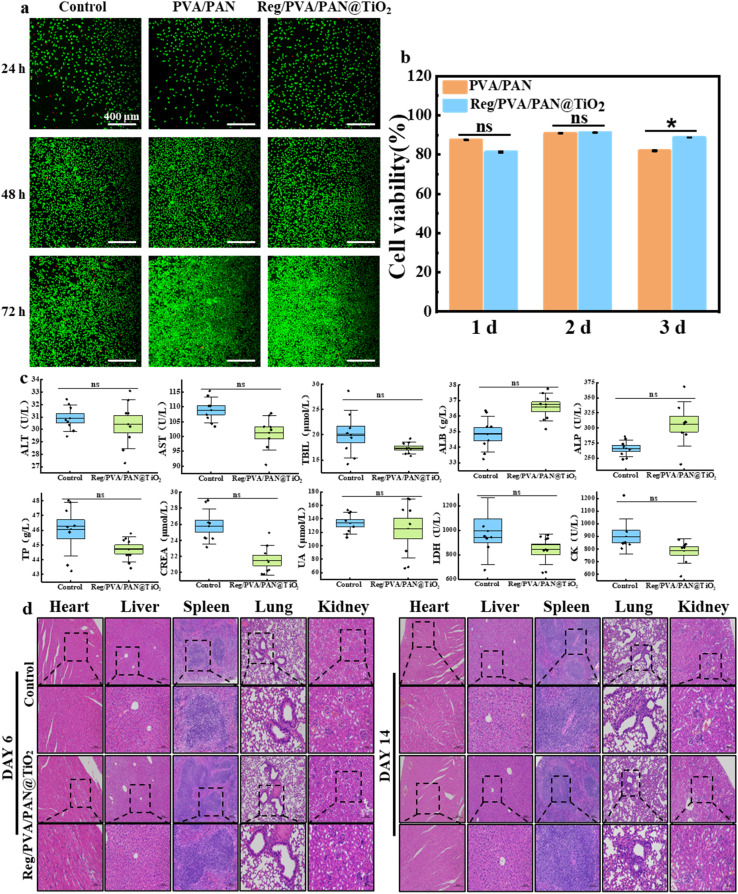
(a) Live/dead fluorescent staining images of L929 cells after incubating with nanofiber dressing at 24, 48 and 72 hours respectively; scale bar: 400 µm. (b) CCK-8 assay of L929 cells after incubating with nanofiber dressing at 24, 48 and 72 hours respectively. (c) Effect of Reg/PVA/PAN@TiO_2_ nanofiber dressing on serum biochemistry. (d) HE staining images of organs in mice (heart, liver, spleen, lung and kidney).

The biocompatibility of PVA/PAN and Reg/PVA/PAN@TiO_2_ nanofiber dressings was systematically evaluated using CCK-8 assays and live/dead staining with L929 fibroblasts (Fig. 6a and b). Both groups exhibited cell viability exceeding 80% after 24–72 h of co-culturing with material extracts (*P* > 0.05), with negligible dead cell ratios observed in fluorescence images. Notably, cell density increased significantly over time (24 h *vs.* 72 h, *P* < 0.01), confirming the non-inhibitory effects of the dressings on fibroblast proliferation. Coupled with their potent antibacterial activity, the Reg/PVA/PAN@TiO_2_ dressing demonstrated a remarkable antibacterial-biocompatible synergy, showing negligible cytotoxicity.

To assess *in vivo* safety, serum biochemistry and histopathology were performed on mice treated with Reg/PVA/PAN@TiO_2_ (Fig. 6c and d, Table S2).The results of the serum markers showed no statistically significant differences (*P* > 0.1) were detected between the experimental and control groups in liver function (ALT, AST, ALP, TP, ALB, TBIL), renal function (CREA, UA), or cardiac enzymes (CK, LDH), ruling out hepatotoxicity, nephrotoxicity, or myocardial injury. At the same time, H&E-stained sections of major organs (heart, liver, spleen, lung, kidney) revealed no structural abnormalities, inflammatory infiltration, or necrosis in either group, further validating the biosafety of the dressing.

## 4. Conclusions

This study successfully developed a Janus-structured Reg/PVA/PAN@TiO_2_ nanofiber dressing through rational material design and electrospinning optimization. Unlike conventional homogeneous materials, each side of the Janus structure has a different chemical composition and microstructure, giving it excellent multifunctional properties through asymmetric synergies and independent mechanisms.

### 4.1 Dual-function structure

The hydrophilic Reg/PVA layer enabled sustained release of RegIIIγ (6% loading), achieving potent antibacterial activity, while the hydrophobic PAN@TiO_2_ layer provided mechanical stability and barrier functionality.

### 4.2 Enhanced wound healing

*In vivo* experiments demonstrated accelerated wound closure (14 days completion), with histopathology confirming structured collagen deposition and reduced inflammation.

### 4.3 Robust biosafety

CCK-8 assays and live/dead staining verified >80% fibroblast viability, while serum biochemistry and organ histopathology ruled out hepatotoxicity, nephrotoxicity, or systemic damage.

In summary, this paper highlights the innovation and application potential of the Reg/PVA/PAN@TiO_2_ nanofiber dressing in wound treatment through its unique bilayer structure design, efficient antibacterial capability, wound healing promotion characteristics, and good biocompatibility. More importantly, this design enhances the biological stability and utilization rate of the antimicrobial peptide. As a result, it provides a novel solution to the problems associated with antimicrobial peptides, such as fast degradation and limited half-life in clinical applications. This brings forward new possibilities for the use of additional antimicrobial peptide medicines.

## Conflicts of interest

There are no conflicts of interest to declare.

## Supplementary Material

RA-015-D5RA05169J-s001

## Data Availability

Data will be made available on request. Supplementary information is available. See DOI: https://doi.org/10.1039/d5ra05169j.
