# Supplementary material for: Janus-structured Reg/PVA/PAN@TiO2 nanofiber dressing containing RegIIIγ recombinant antimicrobial peptides for promoting wound healing
Source: RSC Adv. 2025 Nov 17;15(52):44637–48. doi: 10.1039/d5ra05169j (PMC12622297; doi:10.1039/d5ra05169j)
Supplement: RA-015-D5RA05169J-s001 [file RA-015-D5RA05169J-s001.pdf]

Supplementary Information (SI) for RSC Advances.
This journal is © The Royal Society of Chemistry 2025

Janus-Structured Reg/PVA/PAN@TiO_2 nanofiber dressing containing RegIIIγ recombinant antimicrobial peptides for Promoting Wound Healing

Xuewei Fu [a, 1], Jianrong Chen [b, 1], Xuewen Jian [a], Junkai Wang [c], Minjian Liao [a], Pin Xiong [a], Xiaochun Wu [a], Yuqiao Liu [a], Xianming Dong [a, *], Wuyi Zhou [a, *], and Hui Zhao [a, *]

a. Key Laboratory for Biobased Materials and Energy of Ministry of Education, Research Center of Biomass 3D Printing Materials, School of Materials and Energy, South China Agricultural University, Guangzhou 510642, P. R. China

b. Guangdong Lingnan Health Ecology Technology Group Co., Ltd, Jiangmen 529100, P. R. China

c. School of Veterinary Medicine, South China Agricultural University, Guangzhou 510642, P. R. China

* Corresponding author.

E-mail addresses: totom2008@scau.edu.cn (H. Zhao), zhouwuyi@scau.edu.cn (W. Zhou), dongxming@263.net (X. Dong) , 75843056@qq.com(J. Chen)

[1] These authors contribute equally to this work.

Table of Content

1. Supporting Figures

Fig S1. Plasmid mapping. a pET3a-*RegIIIγ*. b pET32a. c pET32a-*RegIIIγ*.

Fig S2. Expression process of RegIIIγ gene.

Fig S3. Standard curve for protein concentration. The concentration corresponding to the protein was obtained from the standard curve and absorbance calculation of the protein.

Fig S4. a SEM-mapping. (I)SEM images of Reg/PVA, (II) C, (III) O, (IV) N, (V) Cl; b SEM images and diameter distribution.

Fig S5. Tensile poperties and Tensile stress-strain curves of Reg/PVA with different RegIIIγ protein concentrations.

Fig S6. TG curves of nanofiber wound dressings.

Fig S7. Antibacterial activity of the Reg/PVA wound dressings against *S. aureus*.

Table S1. Comparison of the prepared Reg/PVA/PAN@TiO_2 electrospun nanofibers with previously reported wound dressings.

Table S2. Effect of Reg/PVA/PAN@TiO_2 wound dressings on serum biochemistry (mean ± SEM).

1. Supporting Figures

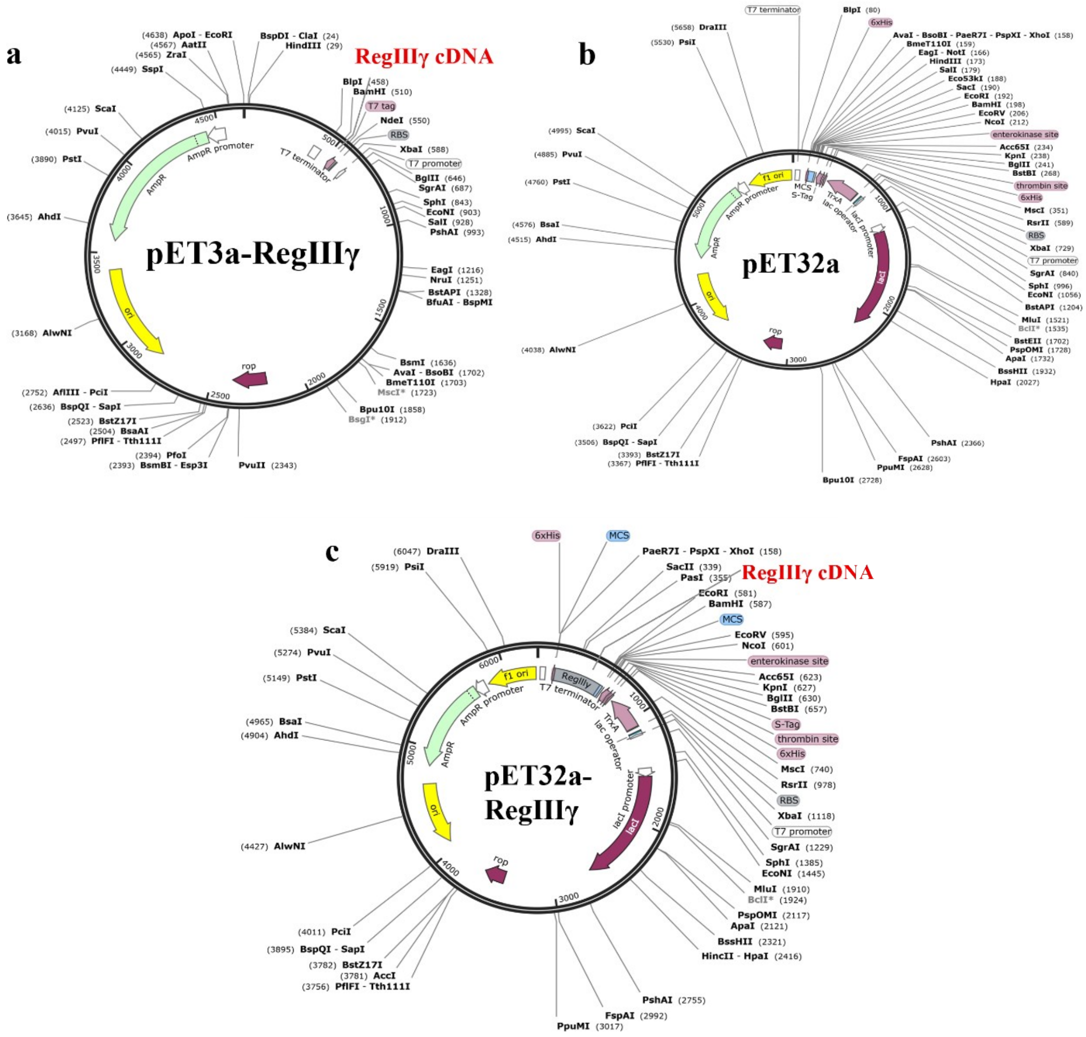

Fig S1. Plasmid mapping. **a** pET3a-*RegIIIγ*. **b** pET32a. **c** pET32a-*RegIIIγ*.

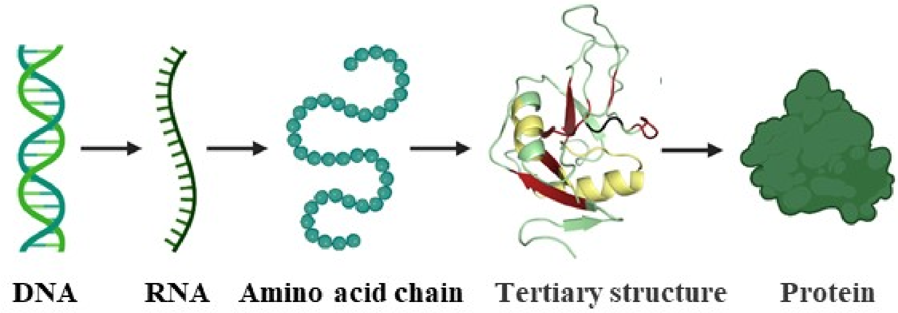

Fig S2. Expression process of RegIIIγ gene.

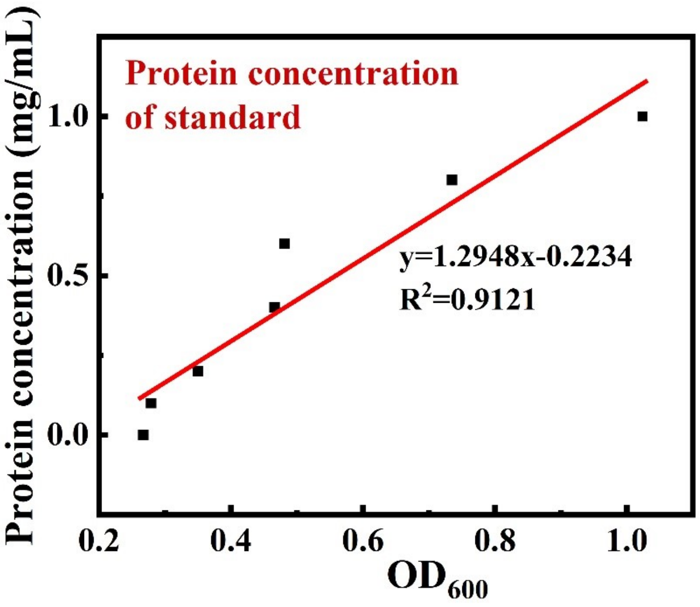

Fig S3. Standard curve for protein concentration. The protein concentration was determined using the standard curve and absorbance calculations.

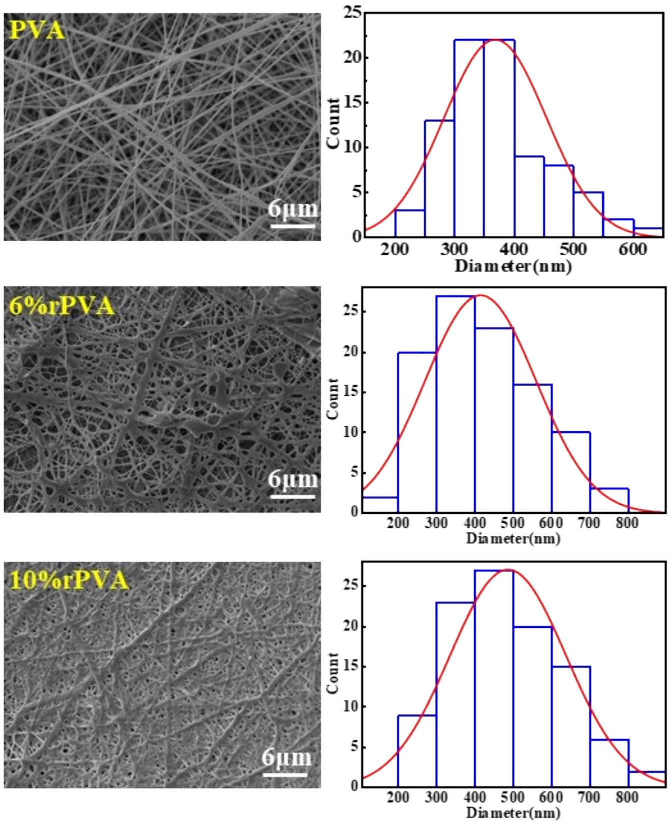

Fig S4. SEM images and diameter distribution.

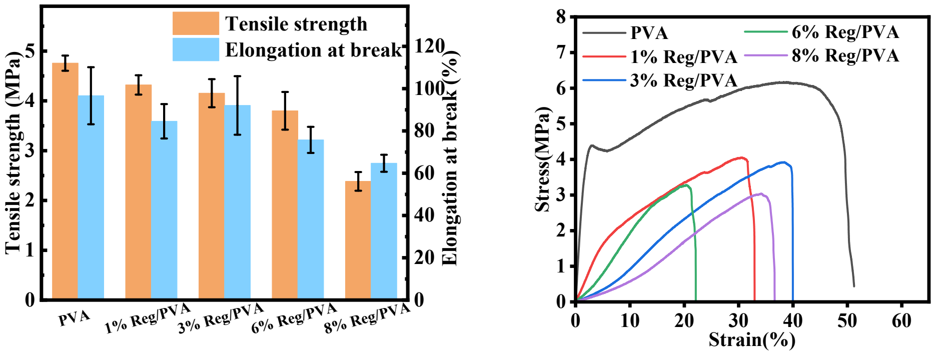

Fig S5. Tensile poperties and Tensile stress-strain curves of Reg/PVA with different RegIIIγ protein concentrations.

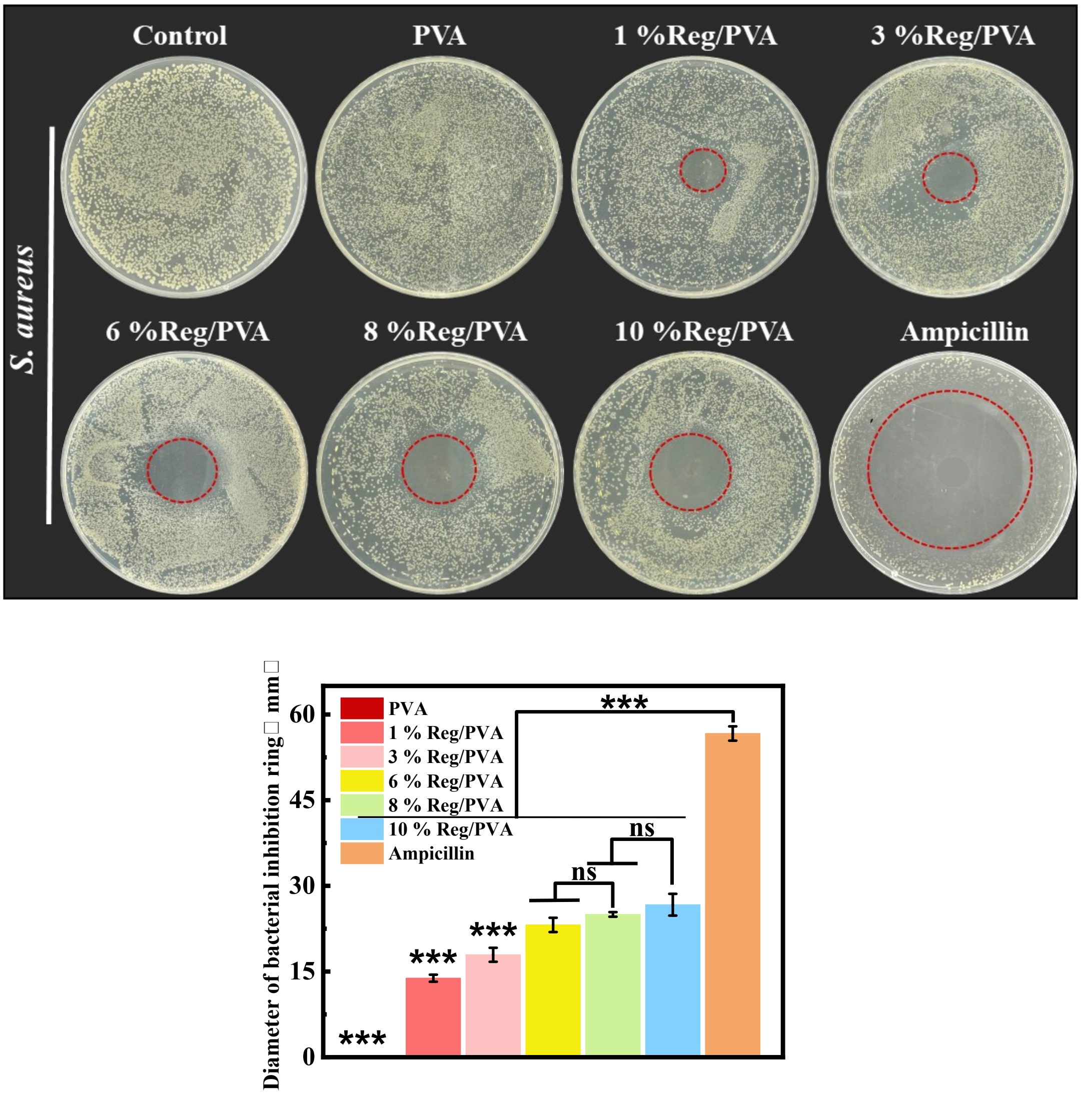

Fig S6. Antibacterial activity of the Reg/PVA wound dressings against *S. aureus*.

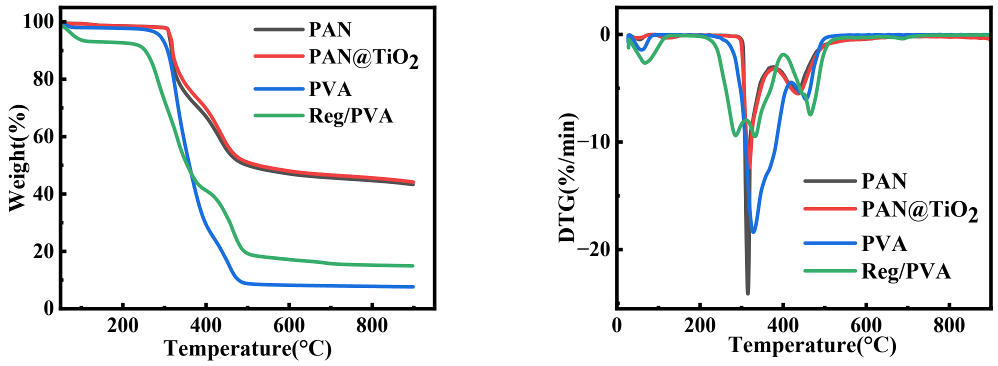

Fig S7. TG curves of nanofiber wound dressings.

Table S1. Comparison of the prepared Reg/PVA/PAN@TiO_2 nanofibrous wound dressings with previously reported wound dressings.

**Components**	**Fabrication method**	**Hydrophilicity or hydrophobicity**	**Antimicrobial activity**	**Cell viability**	**Wound healing rate**	**Tensile Properties**	**Refs.**
PLA/ L-2959	Electrospinning, Hydrogel	Super-hydrophilicity	N/A	85%/24h (NIH/3T3 fibroblasts)	N/A	4Mpa 25%	[39]
CS/PEO/NP10	Electrospinning	N/A	12mm G+/G-	110% (Human red blood cells)	100% 14d	N/A	[40]
BT-OHA/THM-APMH	Hydrogel	Hydrophilicity	95%	90%/72h (NIH/3T3 fibroblasts)	95% 9d	N/A	[33]
NZnO/SF/nHA	Electrospinning	Hydrophilicity	50%	90%/7d (MC3T3-E1)	N/A (bone repair)	2.1Mpa 4%	[41]
PLGA/PDO/ W379	Nanofiber aerogel	N/A	N/A	90%/24h (human keratinocyt-es and HDF)	80% 14d	N/A	[42]
ε -PL/ PCL/Gel	Electrospinning	Hydrophilicity	14mm	OD_{490}=1.1/ 4d (Keratinocyt-es and fibroblasts)	N/A	2.5Mpa 17%	[43]
PDA/PLA/ Gel/SF/NF	Electrospinning, Hydrogel	Hydrophilicity	N/A	OD_{490}=1.2/ 72h (HUVEC cells)	85% 14d	0.106 MPa -0.097 MPa (20th compressio n cycle)	[44]
PLGA/Gel/ PR1P	Electrospinning	Hydrophilicity	N/A	98% (HFF-1 cells)	100% 14d	2.2Mpa 280%	[45]
KGM/BMS / CMS/non-woven	Nanofiber aerogel	Super-hydrophilicity and - hydrophobicity	92%	80%/24h (L929 cells)	100% 14d	N/A	[46]
NIPAM/GTA /HA	Hydrogel	Hydrophilicity	N/A	90%/24h (L929 cells)	98% 21d	N/A	[47]
PCL/PEG /H. perforatum oil	Electrospraying, Electrospining	Hydrophilicity and Hydrophobicity	12mm	110% (L929 cells)	N/A	2.5Mpa	[48]
PCL/PVA/ SSD	Coelectrospinning	Hydrophilicity	4mm/G+	Toxicity effects of SSD	N/A	2.4Mpa 45%	[49]
BTO/PEEK	Electrospinning, coat	Hydrophilicity	96.5%	OD_{value}=0.8/ 72h (BMSC cells)	Ligament-bone healing	N/A	[50]
PVA/PAN/ REGIIIγ/ TiO_2	Electrospinning	Hydrophilicity and Hydrophobicity	28.5mm	90%/48h (L929 cells)	100% 14d	2.5Mpa 35%	Our work

Table S2. Effect of Reg/PVA/PAN@TiO_2 nanofiber dressing on serum biochemistry (mean ± SEM).

Item	Groups		P-value
	Control (n=5)	Reg/PVA/PAN@TiO_2 (n=5)	
ALT（U/L）	30.70 ± 1.31	30.56 ± 2.98	0.944
AST（U/L）	141.87 ± 47.25	101.83 ± 9.83	0.224
TBIL（µmol/L）	22.28 ± 7.40	16.81 ± 1.41	0.277
ALB（g/L）	35.40 ± 1.58	36.59 ± 1.25	0.363
ALP（U/L）	267.54 ± 19.07	306.02 ± 61.50	0.378
TP（g/L）	45.85 ± 2.20	45.00 ± 1.08	0.580
CREA（µmol/L）	61.45 ± 60.41	21.65 ± 2.86	0.318
UA（µmol/L）	129.16 ± 21.15	123.18 ± 52.54	0.864
LDH（U/L）	1078.06 ± 489.90	757.41 ± 175.46	0.346
CK（U/L）	973.81 ± 217.53	697.96 ± 111.21	0.124
